# Genome-wide superior alleles, haplotypes and candidate genes associated with tolerance on sodic-dispersive soils in wheat (*Triticum aestivum* L.)

**DOI:** 10.1007/s00122-021-04021-8

**Published:** 2022-01-05

**Authors:** Darshan Lal Sharma, Roopali Bhoite, Karyn Reeves, Kerrie Forrest, Rosemary Smith, Mirza A. N. N. U. Dowla

**Affiliations:** 1 Department of Primary Industries and Regional Development, 3 Baron-Hay Ct, South Perth, WA 6151 Australia; 2 College of Science, Health, Engineering and Education, Murdoch University, Perth, WA Australia; 3 Centre for AgriBioscience, Agriculture Victoria, Bundoora, AgriBioVIC Australia

## Abstract

**Key message:**

The pleiotropic SNPs/haplotypes, overlapping genes (metal ion binding, photosynthesis), and homozygous/biallelic SNPs and transcription factors (HTH myb-type and BHLH) hold great potential for improving wheat yield potential on sodic-dispersive soils.

**Abstract:**

Sodic-dispersive soils have multiple subsoil constraints including poor soil structure, alkaline pH and subsoil toxic elemental ion concentration, affecting growth and development in wheat. Tolerance is required at all developmental stages to enhance wheat yield potential on such soils. An in-depth investigation of genome-wide associations was conducted using a field phenotypic data of 206 diverse Focused Identification of Germplasm Strategy (FIGS) wheat lines for two consecutive years from different sodic and non-sodic plots and the exome targeted genotyping by sequencing (tGBS) assay. A total of 39 quantitative trait SNPs (QTSs), including 18 haplotypes were identified on chromosome 1A, 1B, 1D, 2A, 2B, 2D, 3A, 3B, 5A, 5D, 6B, 7A, 7B, 7D for yield and yield-components tolerance. Among these, three QTSs had common associations for multiple traits, indicating pleiotropism and four QTSs had close associations for multiple traits, within 32.38 Mb. The overlapping metal ion binding (Mn, Ca, Zn and Al) and photosynthesis genes and transcription factors (PHD-, Dof-, HTH myb-, BHLH-, PDZ_6-domain) identified are known to be highly regulated during germination, maximum stem elongation, anthesis, and grain development stages. The homozygous/biallelic SNPs having allele frequency above 30% were identified for yield and crop establishment/plants m^−2^. These SNPs correspond to HTH myb-type and BHLH transcription factors, brassinosteroid signalling pathway, kinase activity, ATP and chitin binding activity. These resources are valuable in haplotype-based breeding and genome editing to improve yield potential on sodic-dispersive soils.

**Supplementary Information:**

The online version contains supplementary material available at 10.1007/s00122-021-04021-8.

## Introduction

Wheat is one of the most important cereals in human diets and plays a significant role in feeding global population, which is expected to increase by 30% (United Nations 2019) in 2050. The expansion of cultivated land is unlikely due to negative social and environmental impacts (Foley et al. 2011). A continuous increase in wheat demand, amidst climate change and change in soil profiles or fertility will be met by improving crop yield per unit area. Ever since the domestication of wheat about 9000 years back (Peng et al. 2011), progressive selection by farmers and breeders has changed the crop from low yielding locally adapted land races to modern day high yielding cultivars expecting to produce as high as 780 million tonnes in 2021 (FAO forecast 2021). Despite such a high yield potential, realised yield is often low due to production constraints including those posed by soil.

Sodic-dispersive soils comprise multiple subsoil constraints including high pH, salinity, high boron, waterlogging and high aluminium. Dispersive (sodic and magnesic) soils are common in the Australian agricultural landscapes and wheat growing regions around the world. Recent estimates show about 8–10% soils in Western Australian grain belt to be dispersive leading to heavy losses in production and grower income (GRDC fact sheet 2020). The total global area of sodic soils is estimated to be around 830 million hectares, more than 6% of the world’s land (Acosta-Motos et al. 2017; Bennett et al. 2013). Dispersive soils are extremely dense and hinder root growth. The dense structure causes poor movement of air and water infiltration, resulting in low oxygen availability and waterlogging, respectively. These soils have neutral to strong alkalinity (pH 7–12), high content of sodic clay (18%) resulting in high dispersion index (normalised measure of the dispersion of a clay particle in soil. Additionally, the soils contain high concentrations of manganese, boron, aluminium, and salts which restrict root growth and reduce the ability of wheat to extract water and nutrients from relevant layers of soil (GRDC Update 2019). Soil sodicity is assessed as exchangeable sodium percentage (ESP) which in the range of 6–14 corresponds to loss of soil structure by spontaneous clay dispersion and extremely low rates of hydraulic conductivity. Sodic soils severely constrain crop production in Australia and worldwide and ESP as low as 2 can cause notable yield reduction (Sharma 2017). Yield reductions of 70% under sodicity have been reported (Rengasamy 2002). These scenarios highlight the significant loss to agricultural production and the requirement of wheat genetic enhancements to improve yield potential on sodic-dispersive soils.

Tolerance of wheat to sodic-dispersive soils is a quantitative trait, and genetic control is complex. Tolerance is underpinned by several genetic and physiological factors and mechanisms at various wheat developmental stages. Understanding the genetics and mechanisms of sodicity tolerance will enable development of tolerant wheats which will thrive on sodic-dispersive soil. To understand the genetics and address the complex trait improvement, it is a pre-requisite to phenotype yield and yield-components under field conditions. The genetics of sodicity tolerance per se have not been investigated most likely due to its complex genetic effects and inability to mimic the highly variable soil conditions in glasshouse. There are some studies on QTL mapping for soil salinity tolerance identified using glasshouse phenotypic data in cereals (Masoudi et al. 2015; Hussain et al. 2017), but this approach requires extensive validation in alternative genetic backgrounds under appropriate conditions. However, the genome-wide association studies (GWAS) (Turki et al. 2015; Oyiga et al. 2018), using field phenotypic data for diverse lines, can handle this complexity as it incorporates much larger and more representative gene pool, minimising false positives in the estimates of allelic effect. High-density single nucleotide polymorphism (SNP) genotyping arrays are a powerful tool for studying genomic patterns of diversity, inferring ancestral relationships, and studying marker–trait associations in populations (Wang et al. 2014).

Focused Identification of Germplasm Strategy (FIGS) is an effective tool to discover new genes for useful traits in crop plants. It uses machine-learning algorithms to match geographic and agro-climatic characteristics of the site of original collection with target trait to identify desirable genes and alleles (FIGS-ICARDA 2021). The objectives of this study were to (1) use REML/BLUP mixed models to predict true genetic variability of grain yield (YD) and yield-components such as crop establishment/plants m^−2^ (PM), NDVI values (N), heads m^−2^ (HM), grains head^−1^ (GH), 1000 grain-weight (TGW), and harvest index (HI) on sodic and non-sodic soils in FIGS wheat set, (2) conduct GWAS to identify significant and pleiotropic quantitative trait SNPs (QTSs) and haplotypes, contributing to tolerance to sodic-dispersive soils, and (3) identify tolerance associated candidate genes and their functional characterisation, transcription factors and expression status at various wheat developmental stages. The significant and pleiotropic QTSs and haplotypes involved in multi-trait expression and overall grain development will provide information about collaborative gene-networks and genetic mechanisms involved in tolerance on sodic dispersive soils in wheat, which can be deployed in breeding programs and/or genome editing.

## Materials and methods

### Focused identification of germplasm strategy (FIGS) wheat set

The study employed a set of 206 diverse hexaploid wheat lines (Table S1), comprising 190 FIGS lines from 24 countries and 16 Australian check cultivars. These wheat lines from around the world are tolerant to subsoil challenges such as high salinity and pH. Majority of FIGS lines are land races, selected based on the prevalence of subsoil constraints in their place of origin and thus reflect natural selection under target environments (FIGS-ICARDA 2021 and McDonald and Schilling 2019).

### Soil test and experimental design

The experiments were conducted at Dryland Research Institute, DPIRD, Merredin (− 31.499, 118.216), Western Australia, for two years (2018 and 2019). The sodic and non-sodic sites in both years were within 1 km to ensure uniform climatic conditions (temperature, rainfall etc.) for the comparison. The soil type of sodic belongs to clays and shallow loamy duplexes, while the non-sodic site was a deep sandy duplex. Soil analysis for the natural fertility and macro- and micro-nutrients content of both sites from 0–10, 10–30 and 30–70 cm depth is presented in Table 1. A bulk of 10 soil cores of three depth intervals each from experimental site was evaluated for concentration of aluminium (Al), boron (B), copper (Cu), calcium (Ca), iron (Fe), manganese (Mn), magnesium (Mn), phosphorus (P), potassium (K), sodium (Na), sulphur (S), zinc (Zn), exchangeable sodium percentage (ESP), dispersion index, conductivity, organic carbon, and pH. Randomised complete block (RCB) designs were implemented at each site in 2018 and 2019 for FIGS set and Australian control cultivars, with four 4 × 1.5 m^2^ replicate plots for each line. Number of seeds sown for each line was adjusted for germination percentage and seed weight, and a consistent plant density of 120 plants/m^2^ plot was used.

**Table 1:** Soil test data for exchangeable cations in sodic and non-sodic sites for three depth range (0–10, 10–30, and 30–70)

Site	Depth (cm)	Exchangeable cations										Organic carbon^d^	Conductivity^c^	pH	DI	ESP^d^
		K^b^	Ca^a^	Mg^a^	Cu^b^	Zn^b^	B^b^	Mn^b^	Na^a^	Fe^b^	Al^a^					
Sodic	0–10	230.0	3.78	2.38	0.73	0.41	2.46	27.45	0.80	26.6	0.20	0.55	0.07	6.5	9.0	10.6
	10–30	288.0	9.75	7.10	0.92	0.18	7.12	4.50	2.70	12.71	0.18	0.36	0.20	8.7	11.0	13.3
	30–70	300	8.88	8.0	0.95	0.16	20.3	3.49	6.46	10.94	0.19	0.16	0.38	9.4	12.0	24.6
Non-sodic	0–10	115.0	2.21	0.39	0.84	0.67	0.77	11.94	0.07	25.97	0.26	0.62	0.05	5.7	0.0	2.30
	10–30	60	1.88	0.35	0.30	0.17	0.60	4.09	0.06	16.85	0.20	0.26	0.04	6.3	1.0	2.30
	30–70	51	2.20	0.76	0.06	0.40	1.03	0.70	0.15	5.61	0.17	0.12	0.06	5.9	0.0	4.15

Aluminium (Al); boron (B); copper (Cu); calcium (Ca); iron (Fe); magnesium (Mg); manganese (Mn); potassium (K); sodium (Na); zinc (Zn); dispersion index (DI); exchangeable sodium percentage (ESP); units: ^*a*^ (meq100g^−1^), ^*b*^ (mgkg^−1^), ^*c*^ (dSm^−1^), ^*d*^ (%). Bold in the table represents significant difference in metal ion concentration in sodic and non-sodic sites

### Trait measurements and phenotypic data analysis

Yield and yield-components were measured at various wheat developmental stages. Crop establishment/plants m^−2^ (PM) was measured at five-leafed stage (Zadoks GS 13). GreenSeeker NDVI (Model 505, California) values (N) were taken at booting stage (Zadoks GS 49). The instrument was manually held 25 cm above the crop canopy, and each plot NDVI value was averaged over 20 data points. At physiological maturity (Zadoks GS 94), shoot biomass samples from a sub-set of plots (1 m^2^ of 4 × 1.5 m^2^) were collected and dried in an air-forced dryer at 60 °C for 48 h and weighed to estimate biomass per m^2^. These samples were used to assess yield components: heads m^−2^ (HM), grains head^−1^ (GH), thousand grain-weight (TGW), and harvest index (HI). Grain yield (YD) was measured by combine-harvesting the entire experimental plot. Phenotypic data analyses were done using the ASReml-R package (R Core Team 2019) (Butler et al. 2018). A linear mixed model (LMM) was fitted for each trait, using the approach by Lemerle et al. (2006). The model fitted the overall trait means of the sodic and non-sodic as fixed effects and the genotypic effects of the sodic and non-sodic as random effects. The random model accounted for blocking structures of the experiments, heterogeneous genetic variances at the sodic and non-sodic sites, and genetic correlation between the sites. Separate spatial models were applied using the methods by Gilmour et al. (1997) where appropriate. Genotypic effects for each trait in sodic and non-sodic were defined as empirical best linear unbiased predictors and variance components were estimated using residual maximum likelihood (REML) (Patterson and Thompson 1971). Broad-sense heritability (H^2^) was calculated following Bonneau et al. (2013). A relative incremental crop tolerance (ICT) was calculated using trait values from sodic and non-sodic sites, following the method by Lemerle et al. (2006) using the formula, ICT$=Sodic\;blup-(B \times \mathrm{non}-\mathrm{sodic}\;blup)$, where slope B of the regression line is the ratio of the genetic covariance between sodic and non-sodic sites to the genetic correlation at the non-sodic site, B $=cov(\mathrm{non}-\mathrm{sodic }, sodic)/var(\mathrm{non}-\mathrm{sodic })$.

### gDNA extraction

Genomic was extracted from three ground seeds per sample using a CTAB method (Tibbits et al. 2006), replacing isopropanol precipitation with a magnetic bead clean-up. In brief, 120 µl of upper aqueous phase was mixed with 10X diluted AMPure XP beads (Beckman Coulter, USA) in 20% PEG and 2.5 M NaCl, followed by one wash with 200 µl of DNAzol® ES (Molecular Research Centre, USA), that selectively precipitated DNA from a cell lysate. The DNA pellet was washed twice with 70% ethanol and eluted in 15 µl of 10 mM Tris–HCl, pH 8.0.

### Wheat exome tGBS assay and SNP calling

Genotyping was performed by Agriculture Victoria using a targeted genotyping-by-sequencing (tGBS) assay designed to target SNPs evenly distributed across the genome (every ~ 1 Mb) using exome SNP identified from the globally diverse hexaploid wheat collection described in He et al.(2019). Paired-end reads from each sample were trimmed of the GBS probe sequences, concatenated, and filtered to produce a unique list of population-specific allele-specific reference (psASR). Filtering was done to remove allele sequences having a frequency of occurrence < 4 or a minor allele frequency of < 0.25 across samples. The psASR sequences were aligned to IWGSC Chinese Spring v1.0 reference genome assembly (Alaux et al. 2018) using the aligner Nuclear (Gydle Inc.) to identify alleles originating from the same locus to facilitate codominant genotype calling. Genotype calls for individual SNPs are derived from sequence alignment of 180 bp around target SNPs. The high-quality SNP calls for 206 wheat lines were combined to construct a matrix and a hierarchical clustering-based radial dendrogram was produced using R.

### Genome-wide association analyses and linkage disequilibrium of 206-diverse FIGS

GWAS was performed on wheat association panel using phenotypic data collected from two years (2018 and 2019). PCA and kinship analysis was conducted using inbuilt R functions under Genome Association and Prediction Integrated Tool (GAPIT) version 3. The scree plot was used to determine optimum number of principal components (PCs) (Bayesian information criterion) in Q model and kinship heatmap was used to analyze genetic correlation among 206 diverse FIGS wheat lines (Wang and Zhang 2020). Principal components applied to genotypes provide information about population structure and controls type I errors arising from population stratification (Lee et al. 2011). The minor allele frequency (MAF) for the SNP calls was determined (Wang and Zhang 2020; Lipka et al. 2012).

LD among pairs of SNP markers within and across the three wheat genomes (A, B, and D) was estimated as a squared allele frequency correlation (*R*^2^) between SNP marker pairs and the graphical representation of* R*^2^/linkage disequilibrium (LD) against genetic distance (Mb) between the markers was produced using GAPIT package in R.

Association mapping was performed using TASSEL v5 (Bradbury et al. 2007). The high quality 25,471 SNP calls were associated with phenotypic trait BLUP ICTs for two years (2019 and 2019). The pairwise relatedness coefficients (K, kinship matrix) were computed, and the optimal number of principal components (PCs) obtained from scree plot as described above was used to fit the Q model. The MLM (P + K + Q) was tested incorporating the structure (Q) matrix as a fixed factor and the kinship (K) matrix (VanRaden 2008) as a random factor. Quantile–Quantile (Q–Q) plots generated from all the models were compared to select the best model that controls false positives and negatives. To determine whether significant SNP markers associated with the trait on each chromosome were in LD with the highest -log10 (*p-*value) SNP hit, LD analysis was performed on every chromosome where significant quantitative trait SNPs (QTSs) were detected. False discovery rate (FDR) correction (q < 0.10) was performed using Benjamini–Hochberg method to select significant SNPs (Benjamini and Hochberg 1995). Visualisation of the significant QTSs was done using Manhattan plots, generated using the R package qqman (Turner 2018), with chromosome position as the * x*-axis and − log (*p* value) as the * y*-axis.

### Bioinformatics analysis of candidate genes and allele mining

The SNP position on the reference genome was combined with the chromosome number to identify genes and gene ontology associated with sodic-dispersive tolerance. The overlapping genes or genes found close to the significant targeted SNPs (*p* < 0.005; *q* < 0.10) on respective chromosome were searched using BLASTN program, against the Ensembl Plants (Alaux et al. 2018). The Traes numbers of genes with an e-value threshold greater than 10^–85^ and 100% sequence identity were searched in UniProt in TrEMBL (http://www.uniprot.org) and UniParc (https://www.uniprot.org/uniparc/) to obtain more information including protein domain, family, molecular and biological functions of the potential candidate genes. Further, the key features of the domain and InterPro annotation were searched in pfam and Prosite to check the characteristics of the protein. Additionally, alternative splicing variants, variant consequence and SIFT score for the identified genes were determined. The gene ontology (GO) and gene expression levels at various developmental stage were obtained from QuickGo and gene expression atlas, respectively.

## Results

### Soil test and phenotypic variation of yield and yield-components

The soil test comparison of sodic and non-sodic sites revealed higher levels of exchangeable cations on sodic site including toxic levels of boron (B), manganese (Mn) and sodium (Na) and favourable levels of nutrients–potassium (K), calcium (Ca) and magnesium (Mg) (Table 1). However, the two sites had similar levels copper (Cu) and zinc (Zn) and toxic elements–iron (Fe) and aluminium (Al). Dispersion index was 12 times higher in sodic compared to non-sodic site, and pH was above 9.0 at 30–70 cm depth. The phenotypic data for YD, PM, N, HM, GH, TGW, and HI followed normal distribution for both years, indicating multigenic inheritance of the traits. The genetic variance and heritability (H^2^) from the mixed models are presented in Table S2. Data for HM, GH and TGW for 2018 trial are not available. Comparison of the magnitudes of genetic variances at the two soil types (S^2^_non-sodic_ vs. S^2^_sodic_) revealed a greater variation in YD, TGW and HI at the sodic site. Within-year variance comparison for PM and HM showed opposite trends in both years albeit in opposite order; the variance of PM was less than HM on non-sodic than sodic site in 2018 and the vice versa in 2019. Correlation coefficients between trial sites were high and positive for all traits in both years. Moderate to high broad-sense H^2^ was estimated for YD, N, and PM for both the years. However, H^2^ was low for HM, GM, TGW and HI. The plot of sodic blups against non-sodic blups was used to determine ICT for each trait. The slope b in Table S2 was used to calculate ICTs for each trait.

### Genetic diversity of FIGS wheat panel

**Fig. 1:**
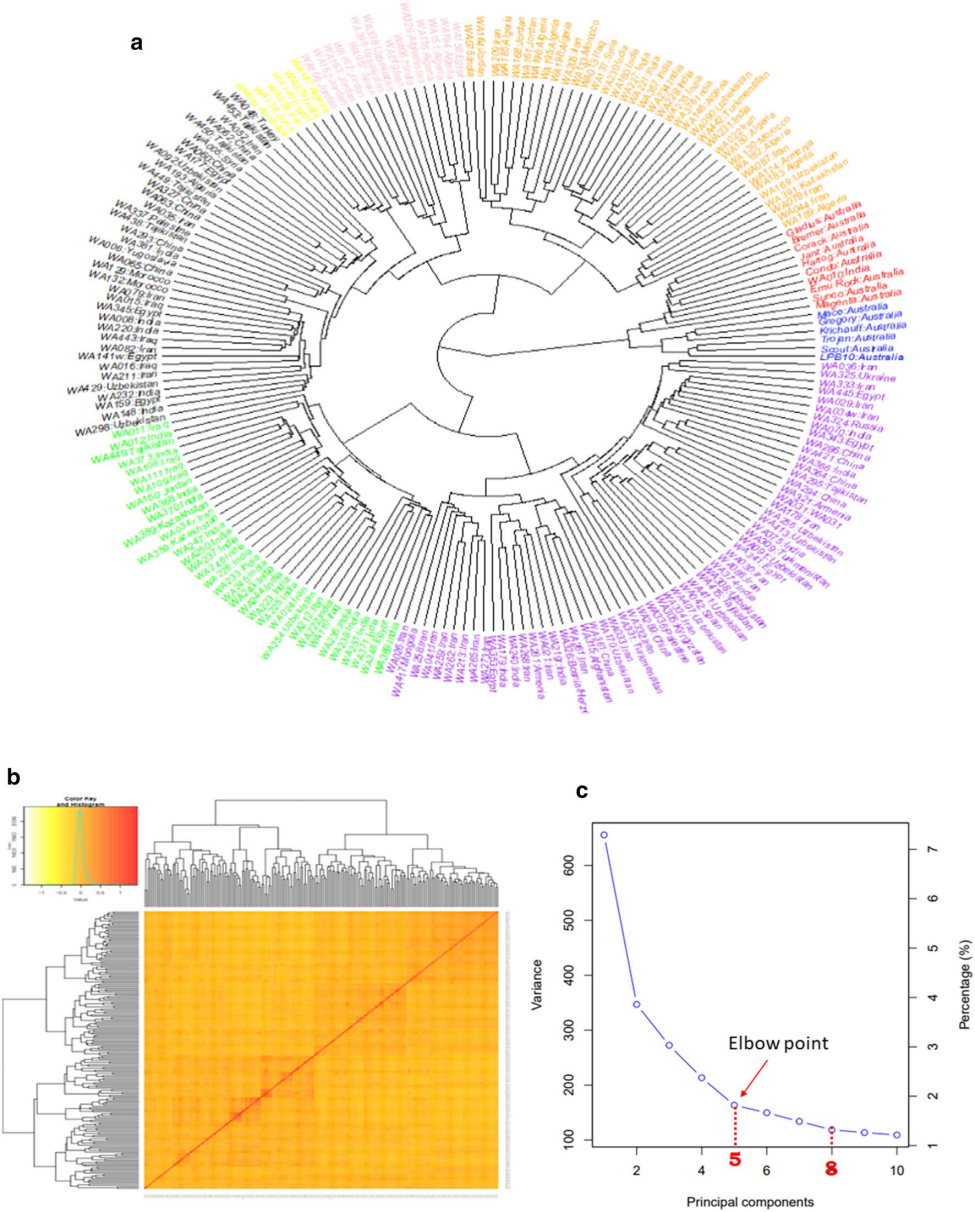
**a** Radial dendrogram elucidating genetic diversity of 206 FIGS wheat lines from 24 countries based on 25,471 SNPs. Colours red, blue, purple, green, black, yellow, pink, and orange indicate eight main clusters **b** Heatmap of relatedness (kinship) among 206 hexaploid wheat lines and dendrogram based on 25,471 SNPs (minor allele frequency ≥ 5%). Dendrograms were plotted using the unweighted-pair-group method with arithmetic mean (UPGMA) clustering. Red colour indicate higher kinship and yellow indicate lower kinship **c** A screeplot from GAPIT showing the selection of PCs/sub-populations based on FIGS diversity panel for association study. The variance reaches the plateau at 5 and therefore the Q model for the present population used 5 principal components to explain the population structure in mixed linear model (colour figure online)

The genetic diversity based in 206 FIGS wheat lines from 24 countries is shown in radial dendrogram (Fig. 1a). The wheat lines from different countries were randomly distributed across eight major clusters, highlighted in eight colours (red, blue, purple, green, black, yellow, pink, and orange), with some of them clustering together, indicating intra- and inter-continental variation. The control check cultivars from Australia were found to be highly related and clustered together. The kinship matrix (Fig. 1b) represented the degree of allele sharing between the FIGS lines. The matrix showed large proportion of yellow, representing low genetic correlation, greater diversity, and an absence of strong population structure among genotypes.

### Linkage disequilibrium, model and significant associations

**Fig. 2:**
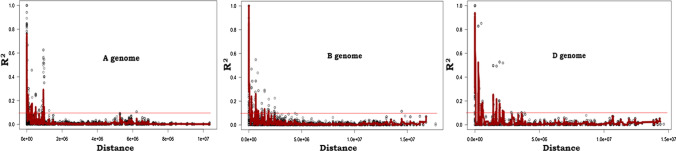
The rate of marker linkage disequilibrium (R^2^) decay of the genome A, B and D of the 206 hexaploid diverse FIGS wheat lines based on 25,471 SNPs

The analysis of linkage disequilibrium (LD) showed that the LD decayed with the genetic distance in A, B and D genomes (Fig. 2). The average of LD in each genome identified the pattern of LD in the three genomes. Based on the average of chromosomes at the genome level, the highest LD was found in the B genome (*R*^2^ = 1.0), followed by genomes D (*R*^2^ = 0.96) and A (*R*^2^ = 0.78). In the present t-GBS assay, D genome (17%) had the lowest number of SNPs followed by genomes A (38.1%) and B (44.8%) (Table S3). The LD decay was rapid in A genome, followed by B and D genome.

The MLM analysis generated a significance value (*P* < 0.005) for each SNP, using *F* test for the null hypothesis that a SNP has no effect on the measured trait. Plots of the negative logarithmic estimates of the observed against expected *p* values (Q-Q plots) showed that the MLM with (Q + K) matrix represented a good model fit and accounted for relatedness between wheat lines, minimising false positives and improving the marker-trait associations estimates (Fig. S1). The scree plot generated by plotting the percentage of variances explained by optimal number of PCs from GAPIT (Fig. 1c) was visualised to select optimal PC number. The elbow point occurred approximately from 5 and therefore, PCs between 5 and 8 were adjusted based on trait variance in Q model. After applying a mixed linear model, we found—927, 1097, 159, 158, 114, 107, and 163 QTNs significantly (*P* ≤ 0.05) associated with ICTs of YD, PM, N, HM, GH, TGW, and HI, respectively. The significant threshold was then adjusted by setting the *P* ≤ 0.005 and q < 0.10. The cut-off threshold *p* values for the traits with FDR correction were YD – 2.9E-03; PM – 1.1E-03; N – 2.2E-03; HM – 5.2E-04; GH – 1.3E-03; TGW – 8.4E-04; HI – 2.6E-03. This reduced the number of significantly associated SNPs to 21 for YD ICT, 18 for PM ICT, 5 for N ICT, 5 for HM ICT, 7 for GH ICT, 5 for TGW ICT, and 12 for HI ICT (Table S4).

### GWAS and potential candidate genes for tolerance to sodic-dispersive soils in wheat

**Fig. 3:**
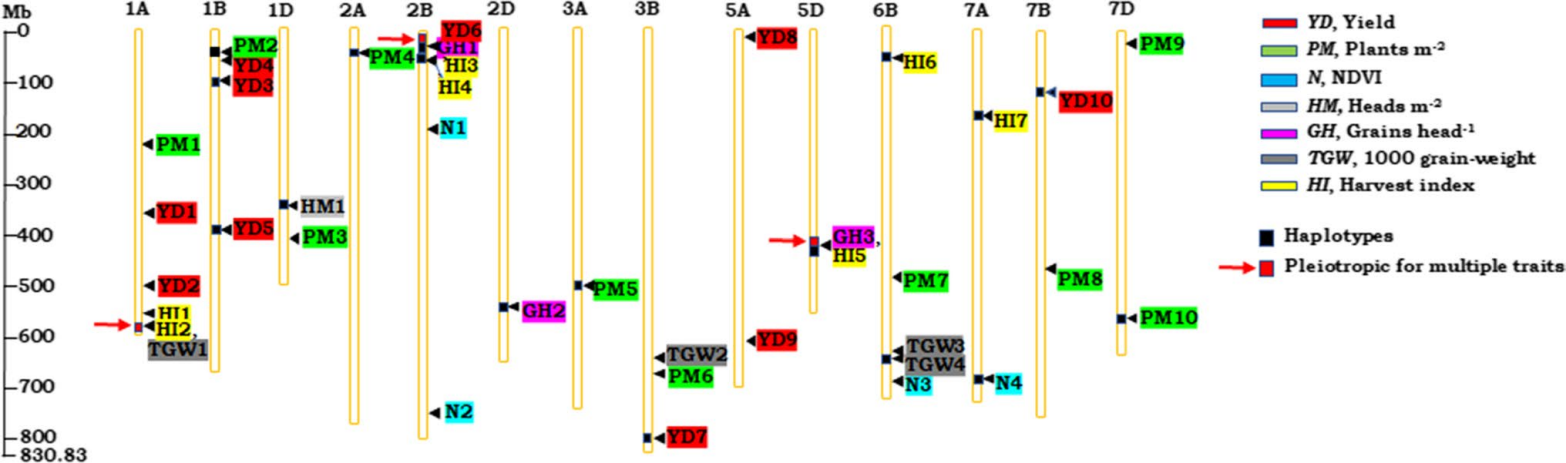
Quantitative trait SNPs identified through association analysis and their respective genomic regions. The trait ICTs are abbreviated: *YD (Red)*, yield; *PM (Green)*, plants m^−2^; *N (Blue)*, NDVI; *HM (Grey)*, heads m^−2^; *GH (Pink)*, grains head^−1^; *SW (Blue)*, 1000 grain-weight; *HI (Yellow)*, harvest index (colour figure online)

A total of 36,644 exome markers were genotyped; of these, 14,925 are co-dominant and 21,719 are dominant. The significant quantitative trait (QTS), Indel, favourable alleles, and their effect identified for two years are presented in Table 2. Targeted SNP and de novo SNP within 180 bp sequence around target SNP were defined as haplotypes. QTS, minor allele frequency and phenotypic variance explained by favourable allele are presented in Table 2. The genome-wide distribution of significant and pleiotropic QTS and haplotypes across 21 wheat chromosomes are shown in Fig. 3. The TRAES genes, length, and location on forward and reverse strand along with its variant consequence, splice variants, GO term, and highest gene expression state at various wheat developmental stage are presented in Table 3. The listed genes are detected either at the exact location of QTS (overlapping) or within 200 Kb from SNP location. The IWGSC low confidence protein coding and uncharacterised genes were not considered for further analysis.

**Table 2:** Significant quantitative trait SNPs (QTSs), haplotypes (H) and favourable alleles contributing tolerance to sodic-dispersive soils identified by genome-wide association analysis

Traits	QTS ^a^	Year	Chr ^b^	Position	Favourable allele	*P*	− log_10_(*P*)	MAF ^c^	PVE (%) ^d^
Yield (YD)	YD1	2019	1A	365,186,774	C	1.3E-04	3.90	0.32	11.8
	YD2	2019	1A	504,980,505	A	2.2E-03	2.65	0.38	4.7
	YD3	2018, 2019	1B	55,014,354	C	2.1E-04	3.68	0.07	5.4
	YD4 **(*H*)**	2018, 2019	1B	94,828,187–94,828,277	A, T, G, A	6.2E-04	3.24	0.07	5.4
	YD5 **(*H*)**	2018, 2019	1B	411,775,338–411,775,423	C, C, C	2.3E-03	2.64	0.42	4.1
	YD6	2019	2B	34,083,794	C	6.3E-04	3.20	0.06	5.2
	YD7 **(*H*)**	2018, 2019	3B	817,580,012–817,580,066	C, C, A	7.0E-05	4.16	0.42	17.8
	YD8	2019	5A	5,713,954	T	1.4E-03	2.86	0.12	4.2
	YD9	2018, 2019	5A	613,704,427–614,358,933	T, A	2.4E-03	2.61	0.07	4.5
	YD10 **(*H*)**	2019	7B	148,015,309–148,015,372	G, T	2.9E-03	2.54	0.12	4.2
Plants m^−2^ (PM)	PM1	2018, 2019	1A	232,828,996	G	1.0E-04	3.98	0.36	39.1
	PM2 **(*H*)**	2019	1B	53,737,652–53,737,716	A, C	2.0E-04	3.69	0.42	14.3
	PM3	2019	1D	420,202,667	T	2.1E-05	4.67	0.33	27.1
	PM4 **(*H*)**	2018, 2019	2A	63,307,274–63,307,383	A, T, T, A, T	2.7E-04	3.56	0.38	24.2
	PM5 **(*H*)**	2019	3A	502,921,633–502,921,779	G, T, T	4.9E-04	3.31	0.35	21.3
	PM6	2019	3B	681,510,374	C	8.3E-04	3.08	0.31	8.1
	PM7	2019	6B	486,517,837	T	3.1E-04	3.52	0.47	37.6
	PM8	2019	7B	484,268,231	T	1.1E-03	2.97	0.15	8.0
	PM9	2019	7D	17,321,139	G	3.1E-04	3.50	0.33	19.8
	PM10 **(*H*)**	2019	7D	566,251,264–566,251,365	C, T	4.7E-05	4.33	0.34	11.6
NDVI (N)	N1	2019	2B	211,009,958	A	1.6E-03	2.79	0.1	7.7
	N2	2019	2B	755,524,000	C	2.2E-03	2.67	0.14	7.2
	N3	2019	6B	694,681,316	A	9.0E-04	3.05	0.47	8.5
	N4 **(*H*)**	2019	7A	696,574,393–696,574,479	A, A	4.3E-04	3.37	0.17	9.1
Heads m^−2^ (HM) Grains head^−1^ (GH)	HM1 **(*H*)**	2019	1D	356,142,294–356,142,422	T, T, G, A, T	5.2E-04	3.29	0.45	9.6
	GH1 **(*H*)**	2019	2B	34,083,794–34,083,886	C, A	1.3E-03	2.91	0.38	5.5
	GH2 **(*H*)**	2019	2D	557,966,612–557,966,723	T, C, G	4.0E-04	3.40	0.09	3.1
	GH3 **(*H*)**	2019	5D	437,358,882–437,358,910	A, A	2.1E-04	3.67	0.24	3.3
1000 grain-weight (TGW)	TGW1	2019	1A	588,006,280	Indel	8.2E-04	3.09	0.02	6.3
	TGW2	2019	3B	658,270,393	G	1.7E-04	3.77	0.03	5.0
	TGW3	2019	6B	638,828,274	C	8.4E-04	3.07	0.34	8.2
	TGW4 **(*H*)**	2019	6B	640,352,471–640,352,516	A, A	5.7E-04	3.24	0.4	8.3
Harvest index (HI)	HI1	2019	1A	555,622,815	A	2.6E-03	2.59	0.04	6.5
	HI2	2019	1A	588,006,280	Indel	3.9E-04	3.41	0.02	6.9
	HI3 **(*H*)**	2018, 2019	2B	34,083,794–34,083,886	C, A	1.9E-03	2.89	0.38	7.2
	HI4	2018, 2019	2B	41,415,831	T	1.5E-03	2.83	0.05	6.7
	HI5 **(*H*)**	2019	5D	437,358,882–437,358,910	A, C	1.2E-03	2.94	0.24	7.0
	HI6 **(*H*)**	2019	6B	69,712,612–69,712,756	C	1.5E-03	2.84	0.05	6.7
	HI7 **(*H*)**	2019	7A	177,135,543–177,135,615	C	6.6E-04	3.18	0.15	7.8

^*a*^ QTS, Quantitative trait SNP; ^*b*^ Chr, Chromosome; ^*c*^ maf, minor allele frequency and MAF < 0.05 are rare variants; ^*d*^ PVE, Phenotypic variance explained; The trait ICTs are abbreviated in QTS: *YD*, Yield ICT; *PM*, Plants m^−2^ ICT; *N*, NDVI ICT; *HM*, Heads m^−2^ ICT; *GH*, Grains head^−1^ ICT; *TGW*, 1000 grain-weight ICT; *HI*, harvest index ICT; haplotypes are denoted as *H*; data for HM, GH and TGW for 2018 trial are not available; Favourable allele locations for haplotypes are presented in supplementary Table S4

**Table 3:** The potential genes identified for QTS, variant consequence, GO term, functional annotation, and highest expression at various wheat development stages

QTS	Chromosome and Location	Gene ID, Length (bp) and direction, and location	Variant consequence	Splice variants	GO term	Wheat developmental stage
YD1	1A (365,186,774)	TraesCS1A02G203400 (2160 +), 115.5 Kb away from QTS	–	2	Brassinosteroid signaling pathway; ATP binding; protein kinase activity	Maximum stem length; anthesis and grain filling stage
YD3	1B (55,014,354)	TraesCS1B02G069500 (1039–), 40.4 Kb away from QTS	–	1	Manganese ion binding; cell wall; apoplast; signal peptide	Germin-like protein – Wheat germination and all developmental stages
YD4 (H)	1B (94,828,187–94,828,277)	TraesCS1B02G093300 (2732 +), overlapping H	Synonymous	2	Nucleic acid binding	Protein SQS1 – Grain development
YD5 *(H)*	1B (411,775,338–411,775,423)	TraesCS1B02G229300 (1174–), 45.8 Kb away from *H*	–	1	Calcium ion binding; apoplast	Calcineurin B-like protein 1 – germination; Grain development
YD6 GH1 *(H)* HI3 *(H)*	2B (34,083,794–34,083,886)	TraesCS2B02G067500 (2797–), 14.8 Kb away from *H*	–	1	Plant secondary metabolism; apoplast	Dirigent protein – Stem elongation
YD7 (H)	3B (817,580,012–817,580,103)	TraesCS3B02G594300 (864 +), 1.7 Kb away from *H*	–	1	RNA metabolism; DNA replication	HTH myb-type domain-containing protein **–** Anthesis
YD8	5A (5,713,954)	TraesCS5A02G008100 (1964 +), overlapping QTS	Intron variant	3	serine-type endopeptidase activity; proteolysis	PDZ_6 domain-containing protein **–** stem elongation, anthesis and grain development
YD9 (H)	5A (613,704,427–614,358,933)	TraesCS5A02G430600 (474 +), overlapping *H*	Missense (SIFT—0.64) S/G€	1	Electron transfer activity	Phytocyanin domain-containing protein **–** Photosynthesis
PM1	1A (232,828,996)	TraesCS1A02G229500 (648 +), overlapping IWGSC low confidence protein coding	–	1	ATP binding; protein kinase activity	Germination; Grain development
PM3	1D (420,202,667)	TraesCS1D02G328700 (1035**–),** 35.0 Kb away	–	1	Chitin binding	Uncharacterized protein
PM4 (H)	2A (63,307,274–63,307,383)	TraesCS2A02G112300 (1454**–**), 51.9 Kb away from *H*	–	1	GTP binding	AIG1-type G domain-containing protein**—**germination; internodes visible stage; anthesis
PM7	6B (486,517,837)	TraesCS6B02G270200 (2903–), overlapping QTS	Intron variant	1	DNA binding and protein dimerisation activity	BHLH domain-containing protein**—**germination; internodes visible stage; stem elongation
N3	6B (694,681,316)	TraesCS6B02G426300 (3013 +), 1.4 Kb away from QTS	–	1	Photoreceptor activity, circadian rhythm, protein ubiquitination	Stem elongation and flowering stage
HM1 **(*H*)**	1D (356,142,294–356,142,422)	TraesCS1D02G262200 (1346 +), overlapping *H*	3 prime UTR variant	1	Metal ion and histone binding, transcription coregulator activity; apoplast	PHD-type domain protein—germination, maximum stem length, flowering opening stage
TGW3	6B (638,828,274)	TraesCS6B02G366100 (1056–), 144 bp away from QTS	–	2	DNA binding, RNA polymerase II cis-regulatory sequence specific binding	Nuclear transcription factor Y subunit—14 days post-anthesis
HI1	1A (555,622,815)	TraesCS1A02G386300 (715 +), 12.4 Kb away from QTS	–	1	Ribonucleoprotein, translation	RPL32—60S ribosomal protein L37a – maximum stem length
HI6 **(*H*)**	6B (69,712,612–69,712,756)	TraesCS6B02G093900 (588 +), 3.6 Kb away from *H*	–	1	Zinc ion and nucleic acid binding; apoplast	Post anthesis, maximum stem length
HI7 **(*H*)**	7A (177,135,543–177,135,615)	TraesCS7A02G213400 (1550 +), 285 bp away from *H*	Missense (SIFT—0.01) V/M^€^	1	Metal and nucleic acid binding; apoplast	Dof-type domain protein—anthesis

Gene ID is the TRAES number according to the URGI-Jbrowse database on Ensembl Plants release; + /– indicates the direction (forward/reverse) on the strand; GO term is the gene ontology; SIFT score is sorting intolerant from tolerant; ^€^ Resulting amino acid: S(serine), G(glycine), V(valine), M(methionine); the trait ICTs are abbreviated in QTS: *YD*, Yield ICT; *PM*, Plants m^−2^ ICT; *N*, NDVI ICT; *HM*, Heads m^−2^ ICT; *GH*, Grains head^−1^ ICT; *TGW*, 1000 grain-weight ICT; *HI*, harvest index ICT; haplotypes are denoted as *H*

**Fig. 4:**
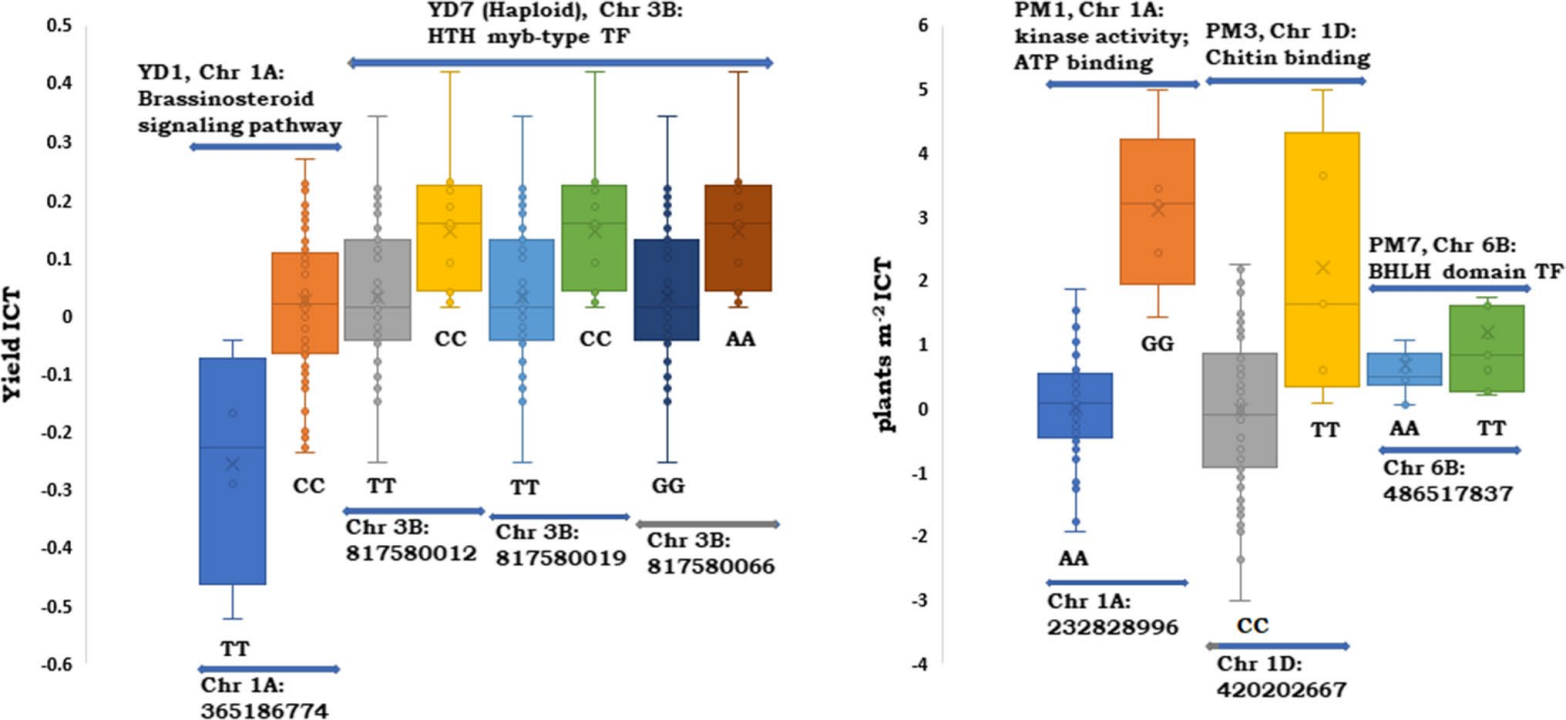
Phenotypic distribution of biallelic SNPs having higher additive marker effect for yield ICT and Plants m^−1^ ICT. The trait ICTs are abbreviated: *YD*, yield; *PM*, plants m^−2^; *TF*, Transcription factor. The Y-axis represents phenotypic ICT values for yield and plants m^−2^, and X-axis represents genomic location of SNPs and haplotypes on wheat chromosomes. The plot represents range of phenotypic values for the alleles at same location for a given SNP

The association analysis for YD ICT revealed strong associations (Table 2) on chromosomes 1A, 1B, 2B, 3B, 5A, and 7B with − log_10_(*P*) value above 2.5 and PVE% in range of 17.8–4.1. Five haplotypes, having more than one SNP within 200 bp on respective chromosome were detected for YD4 (1B): 94,828,187–94,828,277, YD5 (1B): 411,775,338–411,775,423, YD7 (3B): 817,580,012–817,580,066, YD9 (5A): 613,704,427–614,358,933, and YD10 (7B): 148,015,309–148,015,372. The QTSs for YD1 and YD7 are homozygous biallelic and the genotypic effects on yield ICT are presented in Fig. 4. The yield ICT increased by 11.3% in the presence of beneficial allele C with respect to allele T at YD1 (365,186,774) on chromosome 1A. Similarly, for haplotype YD7, the beneficial allelic combination CC, CC, and AA at locations 817,580,012, 817,580,019 and 817,580,066, improved average yield ICT by 79.5% with respect to the allele TT, TT and GG, respectively. In general, most of the overlapping genes for YD ICT have activities for metal ion binding, DNA and ATP binding, proteolytic, and electron transfer. These genes have highest expression during grain development, anthesis, stem elongation, photosynthesis, and germination.

The association analysis for PM ICT revealed ten QTSs on chromosomes 1A, 1B, 1D, 2A, 3A, 3B, 6B, 7B and 7D, with − log_10_(*P*) value above 3.0 and PVE% in the range of 39.1–8.0. Four haplotypes were detected on QTSs PM2 (1B): 53,737,652–53,737,716, PM4 (2A): 63,307,274–63,307,383, PM5 (3A): 502,921,633–502,921,779, and PM10 (7D): 566,251,264–566,251,365. Two QTSs PM4 (2A) and PM7 (6B) had significant impact on wheat establishment on sodic-dispersive soil with PVE% of 24.2 and 37.6, respectively. The QTSs PM1, PM3 and PM7 are homozygous biallelic having higher additive effect for wheat establishment (Fig. 4). The presence of the allele GG (232,828,996), TT (420,202,667) and TT (486,517,837) at PM1, PM3 and PM7, improved average wheat establishments by 85%, 99% and 43.7% with respect to alleles AA, CC and AA, respectively. Most of the overlapping genes identified for PM ICT have protein kinase activity and binding activities for ATP, chitin, GTP, DNA, and protein dimerisation. These genes have highest expression during germination, internodes visible stage, and anthesis. The association analysis for N ICT identified four QTSs on chromosomes 2B, 6B and 7A with − log_10_(*P*) value above 2.5 and PVE% in the range of 9.1–7.2. A haplotype was detected on chromosome 7A at QTS N4 (7A): 696,574,393–696,574,479. The QTS N3 on chromosome 6B at 694,681,316 had minor allele frequency of 0.47 with PVE% of 8.5 and this QTS corresponds to a gene TraesCS6B02G426300, having photoreceptor activity, highly expressed during stem elongation and flowering stage.

The association analysis for yield-components ICT of HM, GH, TGW, and HI identified 1, 3, 4, and 7 QTSs, respectively. All QTSs had − log_10_(*P*) value above 2.5 and PVE% in the range of 9.6–3.1. A haplotype was identified for HM ICT on chromosome 1D (HM1). This haplotype has five SNPs between 356,142,294 and 356,142,422, which is within 128 bp with a minor allele frequency of 0.45 and PVE% of 9.6. This genomic region overlaps with the gene TraesCS1D02G262200, involved in metal ion binding and transcription regulation, and is highly expressed during germination, flower opening stage and maximum stem elongation. Three haplotypes were identified for GH ICT on chromosome 2B, 2D and 5D. The haplotype on chromosome 2B overlaps with the gene TraesCS2B02G067500, which is pleiotropic for YD and HI and encodes for dirigent protein, highly expressed during stem elongation. Likewise, four QTSs were identified for TGW ICT on chromosome 1A, 3B and 6B. The QTS TGW3 on chromosome 6B at 638,828,274 corresponds to a gene TraesCS6B02G366100 which has two splice variants and is involved in RNA polymerase II cis-regulatory sequence specific binding. This gene is highly regulated 14 days after anthesis. The haplotype QTSs TGW4: 640,352,471–640,352,516, is 1.52 Mb apart from TGW3: 638,828,274 on same chromosome 6B. Seven QTSs were identified for HI ICT on chromosome 1A, 2B, 5D, 6B, and 7A. The haplotypes were identified on chromosome 2B (34,083,794–34,083,886), 5D (437,358,882–437,358,910), 6B (69,712,612–69,712,756), and 7A (177,135,543–177,135,615). The overlapping genes on chromosome 1A, 6B, and 7A have metal ion and nucleic acid binding and translation activity and these genes are highly expressed during anthesis, post-anthesis and maximum stem length. The gene TraesCS7A02G213400 caused missense mutation with a deleterious SIFT score of 0.01, inducing an amino acid change from valine to methionine. Most of the QTSs identified for TGW and HI ICT had rare variants MAF < 0.05 (Table 2).

### Pleiotropy of Quantitative trait SNPs

Genome-wide analysis for tolerance to sodic-dispersive soil revealed that three regions contained QTSs for more than one trait ICT (Fig. 3 and Table 2) at same genomic region representing pleiotropy. Chromosome 1A: 588,006,280 harboured QTS for TGW (TGW1) and HI (HI2) at same genomic location. Chromosome 2B: 34,083,794 harboured QTS for YD (YD6), GH (GH1), and HI (HI3). QTSs with haplotypes on chromosome 2B, GH1 and HI3 had two SNPs located within 92 bp (34,083,794–34,083,886) and are pleiotropic. A haplotype in chromosome 5D: 437,358,882–437,358,910, harboured two QTSs detected within 28 bp interval, having common associations for GH (GH3) and HI (HI5), indicating pleiotropism.

Additionally, the closely related association for traits can be visualised in Fig. 3. The QTS for HI on chromosome 1A, HI1 (555,622,815) is found close to HI2 (588,006,280) and TGW1 (588,006,280), within 32.38 Mb. The QTS for YD on chromosome 1B, YD3 (55,014,354) is found close to haplotype PM2 (53,737,652–53,737,716) within 1.27 Mb. The QTS for HI on chromosome 2B, HI4 (41,415,831) is found close to YD6 (34,083,794), GH1 haplotype (34,083,794–34,083,886) and HI3 (34,083,794–34,083,886), within 7.33 Mb. The QTS for TGW on chromosome 3B, TGW2 (658,270,393) is found close to PM6 (681,510,374), within 23.23 Mb.

## Discussion

Sodic-dispersive soils have a compact soil structure, often accompanied by high levels of metal ion concentration, pH, bulk density, dispersion index, and salinity (Table 1), which constrain root and shoot growth, biomass accumulation and ultimately grain yield. Sodic soils have high clay content, and the exchangeable cations attach to the clay particles. Under moist condition, large amount of clay particles tend to separate from each other causing dispersion and breakdown of soil aggregates. The contribution of different ions to net dispersion depends upon their relative proportion, physical size, and ionic charge, where Na^+^ have the most detrimental effect. The dispersed clay clogs the soil pores resulting in soil crusting and loss of soil structure, which significantly impacts crop germination, root growth and water and nutrient uptake. This condition is most prevalent during heavy rainfall events in the start of cropping season in Australia. Although nutrients such as boron contribute to overall plant growth, they can become toxic in excess, which is problematic. The phenotypic data on flag leaves in wheat have shown metal toxicity for B and Al (GRDC Update, 2019) on sodic-dispersive soils. Accumulation of high levels of metal in plants contributes to the generation of reactive oxygen species (ROS), which can cause extensive cellular damage. Investigating the causes of genetic variation and understanding how these chemicals interact in sodic-dispersive soils will help in developing tolerant cultivars.

High genetic variability for sodic-dispersive soil tolerance was found for yield and yield-components. For the first time, a detailed phenotyping incorporating yield and yield-components under sodic and non-sodic soil types was recorded. The considerable differences in genetic variances of YD at the sodic and non-sodic for both the years (Table S2) confirmed the suitability of testing conditions for phenotypic differentiation of tested germplasm. The opposite trend of within-year variance comparison between the two sites for PM and HM (comparison available only in 2019), aligned with the concept of compensation for yield components in setting sink size (Sharma et al. 2008; Anderson et al. 2011; Slafer et al. 2014). Occurrence of high heritability for yield but lower heritability for yield components indicated high plasticity of yield components. The high and positive correlation coefficients between trial sites reflected an overall similarity of the yield structure and background confounding of tolerance with yield levels. The genetic variance of YD and HI tolerance was more apparent in 2019 compared to 2018. This reflects an overall variance compaction due to reduced mean values from the harsher seasonal conditions in the start and end of the crop season in 2019. The average seasonal rainfall during early cropping season (April to October) was less in 2019 (208 mm) compared to 2018 (230 mm). However, year 2019 had heavy rainfall events soon after sowing which caused serious soil crusting and weak germination. Likewise, low rainfall events during mid-late cropping season had severe effects on grain filling and yield in 2019.

### Genetic diversity, LD and mixed linear model

The wide genetic diversity and population clusters mostly results from geographic and gene exchange isolation, and this aspect is vital in genome-wide association analysis (Wen et al. 2018; Huang et al. 2017). Genome-wide association analyses in a largely diverse FIGS panel revealed genome-wide significant SNPs for yield and yield-components (Table S4). The population structure (Q) and kinship (K) are the common confounding factors in GWAS. The MLM (P + Q + K) incorporating the association between genotypes (SNPs), phenotypes (P), population structure (Q matrix) and kinship (K matrix) for two years minimised the false-positives (Bradbury et al. 2007) and uncovered the genetic basis of complex yield and yield-components contributing to tolerance to sodic-dispersive soils.

Investigating the LD structure enables identification of the genomic regions having large genetic effects on complex trait expression and determining the density of markers needed to associate the genotypes with the traits. LD, the non-random association of alleles at different loci, plays an integral role in association mapping, and the LD magnitude and decay determines the resolution of association study (Flint-Garcia et al. 2003). The extent of LD differs across genomes in many species, and wheat is an allohexaploid containing three different genomes A, B and D.

The LD decay distance provides an estimate of recombination rate. The greater the recombination rate, the smaller the LD decay distance and greater the need for higher marker density to achieve high mapping resolution. Higher the LD decay distance, there is less possibility of recombination events within the stretch and there are high chances that the QTSs and haplotypes are co-inherited. In the present diverse FIGS population, LD decayed in genome D at a higher genetic distance than genome A and B, which suggested that fewer markers are sufficient to detect target QTSs on genome D using GWAS than the other two genomes (Liu et al. 2017). In the present investigation, D genome had the lowest number of SNPs followed by genomes A and B, which suggest that current SNPs considered for association mapping are appropriate to identify significant alleles associated with tolerance to sodic-dispersive soils.

### Association analysis and candidate genes for tolerance to sodic-dispersive soils

The association analysis for tolerance on sodic-dispersive identified QTSs, pleiotropic SNPs, haplotypes, overlapping genes and transcription factors contributing to tolerance to sodic-dispersive soils. QTSs were identified on chromosome 1A, 1B, 1D, 2A, 2B, 2D, 3A, 3B, 5A, 5D, 6B, 7A, 7B, 7D. Group 1, 2 and 7 chromosomes have largely contributed to tolerance to sodic-dispersive soils. The QTSs corresponding to metal binding and photosynthetic genes identified at different developmental stages largely represent genes having cumulative genetic effect in expressing tolerance to sodic-dispersive soils in wheat. SNPs with rare variants (MAF < 0.05) (Table 2) usually appear more frequently in coding regions than common variants (MAF > 0.05) (Herandez et al. 2019; Sidore et al. 2015). These rare variants may represent favourable mutations in the population, contributing to trait expression and play an important role in heritability. However, the effects of rare variants on trait expression need to be validated.

Phytohormones, protein kinase and chitin-binding activities play a significant role in alleviating stress in sodic-dispersive soils. The present association study revealed significant additive effects with greater phenotypic variance (PV) for the QTS corresponding to phytohormones, kinase and chitin binding activities (Table 2). The QTS YD1 on chromosome 1A, corresponds to the gene involved in brassinosteroid (BR) signaling pathway. BR-responsive genes drive cellular growth, plant adaptation to environmental stress, early flowering, and increased grain yield in wheat (Singh et al. 2016). The overlapping gene for QTSs YD5 and PM1 encodes calcineurin B-like protein (CBL) and protein kinase (PK), respectively, highly expressed during germination and grain development (Table 3), having important role in abiotic stress responses, and calcium ion exclusion (Batistic and Kudla, 2008). The overexpression of CBL and PK showed a higher survival rate under abiotic stress with enhanced germination rate, well developed root system, increased accumulation of osmolytes, and reduced rate of water loss (Cui et al. 2018). Similarly, the QTS PM3 corresponds to the genes encoding chitin-binding activity. Chitin-binding proteins encoded by chitin-gene family enhance resistance to different stresses in crop plants (Ali et al. 2018).

The sodic-dispersive sub-soils have high levels of exchangeable boron and manganese cations (Table 1). The metal binding genes reported in this study promote diffusion of metals through apoplast thus preventing metal toxicity. The QTS YD3 on chromosome 1B overlaps gene encoding germin-like protein, which is a signal peptide involved in abiotic-stress responses, including manganese (Bernier and Berna, 2001), aluminium, salt, submergence, and heat in wheat and barley (Patnaik and Khurana, 2001; Dunwell et al. 2000). Additionally, they play a significant role during germination and various wheat developmental stages. Exclusion of aluminium ions in apoplast has prevented toxic levels, improving shoot growth and crop yield in higher plants (Horst and Walter, 1995).

Plant-specific transcription factors play a critical role in plant growth and development. The metal ion binding genes identified for QTSs HM1 and HI7 encodes for PHD-type domain protein and Dof-type domain proteins, respectively, which has transcriptional regulatory activities for plant growth and development (Sanchez and Zhou 2012; Yanagisawa 2004). Interestingly, the Dof-type domain protein gene TraesCS7A02G213400 caused deleterious missense variant (SIFT – 0.01). The biallelic SNPs on chromosome 3B (YD7) and chromosome 6B (PM7) with greater additive effects, explaining greater phenotypic variance corresponds to transcription factors, HTH myb-type and BHLH-domain containing protein, which regulates replication and transcription during germination, stem elongation and anthesis. Similarly, QTS TGW3 on chromosome 6B corresponds to nuclear transcription factor Y sub-unit which is involved in transcription. MYB-type and BHLH-domain transcription factors are involved in tolerance mechanisms of several abiotic stress and usually co-expressed to enhance grain size under abiotic stress in cereal crops (Bhoite et al. 2021; Watt 2020; Roy 2015). The MYB-type transcription factor regulated cadmium tolerance by preventing oxidative damage in Arabidopsis (Agarwal et al. 2020), and transcript level of MYB-type transcription factor was enhanced in response to drought stress in potato (Shin et al. 2011). The PDZ_6 domain-containing protein encoded by TraesCS5A02G008100, present on chromosome 5A, has three spliced transcript variants and is involved in the coordination of a diverse range of regulatory processes for ion transport and multi-drug resistance. RPL32 identified for HI1 on chromosome 1A is a protein coding gene which is highly expressed for maximum stem length (Wheat RefSeq, 2018 – URGI portal) (Alaux et al. 2018).

The photosynthetic genes play a vital role in all developmental stages. The gene TraesCS5A02G430600 identified for YD9 on chromosome 5A, encodes phytocyanin domain protein involved in photosynthesis. Phytocyanins (PCs) are plant-specific blue copper proteins involved in electron transport (Cao et al. 2015). The function of PCs in stress responses of protecting cell from aluminium toxicity has been reported (Ezaki et al. 2001, 2005). This gene causes a missense variant, inducing amino acid change from serine to glycine. However, the SIFT score of 0.64 does not imply deleterious effect. Other genes related to photosynthesis are involved in GTP binding (TraesCS2A02G112300) which directs proteins to thylakoid membrane and photoreceptor activity (TraesCS6B02G426300) that senses a light signal and transmits it to the circadian clock. These genes significantly control yield and yield-related traits (Sun et al. 2020). Further, we aim to validate candidate genes and analyse gene functions using virus-induced gene silencing (VIGS)—a powerful functional genomics tool to validate the candidate genes (Kumar and Kiran Kumar, 2011; Becker and Lange 2010) for crop improvement programs.

### Relationship between yield and yield-components for tolerance to sodic-dispersive soils

The study identified pleiotropy QTSs/loci for multiple traits, on Chromosome 1A: 588,006,280 for ICTs of TGW and HI; Chromosome 2B: 34,083,794–34,083,886 for ICTs of YD, GH and HI; chromosome 5D: 437,358,882–437,358,910 for ICTs of GH and HI and other close associations for yield and yield-components (Fig. 3). Given that yield is the net outcome of yield components (Calderini et al. 1995), the pleiotropy evident in this study was more often associated with YD, GH and HI. The gene overlap within the reported pleiotropic range has a major genetic effect for expressing tolerance to sodic-dispersive soils. The candidate gene TraesCS2B02G067500 identified for traits—YD, GH and HI on chromosome 2B encodes for dirigent proteins, predominantly expressed in stems (Li et al. 2017), having roles in biosynthesis of lignin-like molecules, defence responses, secondary metabolism, and fibre synthesis. Many dirigent genes are inducible by abiotic and biotic stress factors (Wu et al. 2009), however, the role of dirigent proteins in metal ion exclusion is unknown.

## Conclusion

This is the first report on GWAS for tolerance on sodic-dispersive soils in wheat. The study reported that sodic-dispersive tolerance in wheat is a complex trait governed by many genes at various developmental stages. The study identified potential genomic regions, haplotypes, pleiotropic SNPs, overlapping genes and transcription factors governing yield and yield-components. The homozygous/biallelic SNPs (YD1, YD7, PM1, PM3, and PM7) with greater additive effects explaining large phenotypic variance (PVE% > 10) were identified which may contribute significantly to the yield and wheat establishments on sodic-dispersive soils. The in silico gene expression analyses revealed that genes are highly expressed during germination, stem elongation, anthesis, and grain development and are involved in metal ion exclusion, photosynthesis, and transcription regulation. The pleiotropic SNPs, haplotypes, transcription factors and overlapping genes identified in the present study provide new potential targets for genetic improvement of wheat on sodic-dispersive soils.

In most crop genomes, the exome corresponds to only 1–2% of the entire genome. The gene-associated t-GBS platform had SNPs tagging key trait loci in A, B and D genomes of wheat. The array served as an invaluable resource for inferring significant and pleiotropic QTSs, transcription factors, homozygous/biallelic SNPs with significant additive effects contributing to the trait expression, overlapping genes with expression status at various wheat developmental stages, and haplotype diversity in a globally diverse wheat germplasm. The common and distinct haplotype detected for several agronomic trait expression could simplify study of the genetic basis of complex tolerance expression to sodic-dispersive soils. It can be noted that most of the candidate genes identified for yield and yield-components have higher expressions during germination and early growth stages and these SNPs can be deployed in crop improvement programs. The significant homozygous biallelic codominant markers reported in the present study can be potentially converted to KASP markers for high throughput marker-assisted selection, reducing time, cost, and error rates. Alternatively, CRISPR base-editing tool can be used to bring about favourable allele editing, improving the tolerance and yield potential on sodic-dispersive soils.

## Supplementary Information

Below is the link to the electronic supplementary material.

Supplementary file1 (XLSX 14 KB)

Supplementary file2 (DOCX 104 KB)
