# Supplementary material for: Genome-wide superior alleles, haplotypes and candidate genes associated with tolerance on sodic-dispersive soils in wheat (Triticum aestivum L.)
Source: Theor Appl Genet. 2022 Jan 5;135(3):1113–28. doi: 10.1007/s00122-021-04021-8 (PMC8942925; doi:10.1007/s00122-021-04021-8)
Supplement: Supplementary file 2 — Supplementary file2 (DOCX 104 KB) [file 122_2021_4021_MOESM2_ESM.docx]

**
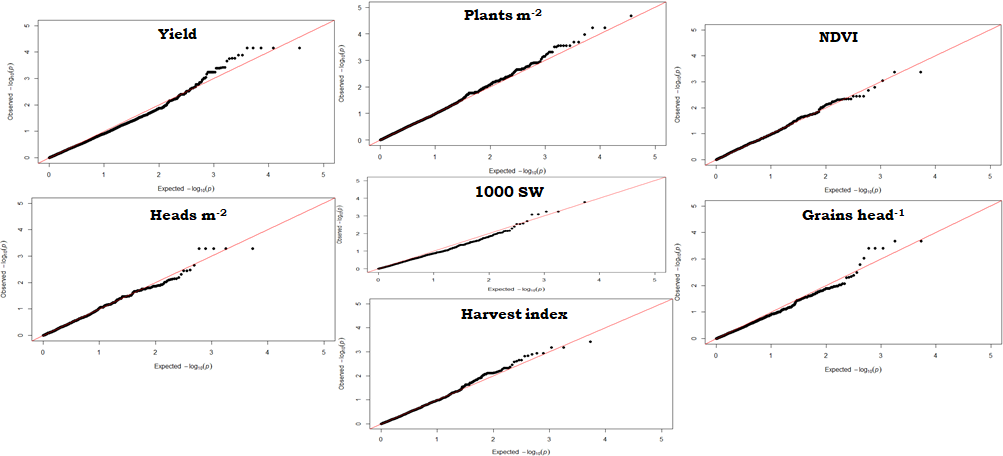
**

**Figure S1** Quantile-quantile plots between expected and observed *p* values (–log10­) for the trait ICTs of yield, NDVI values, plants m^-2^, heads m^-2^, grains head^-1^, 1000 seed weight and harvest index. The straight diagonal lines are the reference correlation.

**Table S2** Genetic **v**ariance parameters and heritabilities of sodic and non-sodic phenotypic data and incremental crop tolerance

| Trait | Year | S^2^_NS_ | S^2^_SD_ | r_SD,NS_ | H^2^_NS_ (%) | H^2^_SD_ (%) | b | S^2^_ICT_ |
| --- | --- | --- | --- | --- | --- | --- | --- | --- |
| Yield (YD) | 2018 | 0.07 | 0.095 | 0.859 | 82.0 | 85.0 | 1.000 | 0.024 |
|  | 2019 | 0.096 | 0.262 | 0.926 | 89.9 | 93.4 | 1.532 | 0.037 |
| NDVI (N) | 2018 | 0.0066 | 0.006 | 0.795 | 70.3 | 44.1 | 0.756 | 0.0022 |
|  | 2019 | 0.0032 | 0.0028 | 0.943 | 69.3 | 69.0 | 0.881 | 0.0003 |
| Plants m^-2^ (PM) | 2018 | 1977 | 243.3 | 0.947 | 87.1 | 82.3 | 0.332 | 25.18 |
|  | 2019 | 144.2 | 166.8 | 0.952 | 63.4 | 64.2 | 1.020 | 15.51 |
| Heads m^-2^ (HM) | 2019 | 2563 | 860 | 0.196 | 56.3 | 53.0 | 0.114 | 827 |
| Grains head^-1^ (GH) | 2019 | 17.64 | 26.71 | 0.931 | 71.8 | 75.3 | 1.146 | 3.53 |
| 1000 seed weight (SW) | 2018 | 17.95 | 20.38 | 0.879 | 34.4 | 35.8 | 0.936 | 4.64 |
|  | 2019 | 19.36 | 27.05 | 0.954 | 90.6 | 93.1 | 1.128 | 2.409 |
| Harvest index (HI) | 2018 | 0.0021 | 0.0025 | 0.940 | 30.3 | 31.4 | 1.023 | 0.0003 |
|  | 2019 | 0.0031 | 0.0039 | 0.846 | 46.1 | 82.4 | 0.951 | 0.0011 |

S^2^_NS_: Estimated genetic variance non-sodic; S^2^_SD_: Estimated genetic variance sodic; r_SD,NS_: Correlation coefficient; H^2^_NS_ (%): Estimated heritability non-sodic; H^2^_SD_ (%): Estimated heritability sodic; ICT: Incremental crop tolerance; b: The beta used in the calculation of ICT; s^2^_ICT_: Estimated genetic variance of ICT

**Table S3** The SNP densities across the 21 wheat chromosomes

| Chr^a^ | 1A | 1B | 1D | 2A | 2B | 2D | 3A | 3B | 3D | 4A | 4B | 4D | 5A | 5B | 5D | 6A | 6B | 6D | 7A | 7B | 7D | UN |
| --- | --- | --- | --- | --- | --- | --- | --- | --- | --- | --- | --- | --- | --- | --- | --- | --- | --- | --- | --- | --- | --- | --- |
| No. of SNP sites | 1417 | 1764 | 606 | 1618 | 2000 | 1055 | 1113 | 1696 | 607 | 1436 | 972 | 317 | 1341 | 1881 | 606 | 939 | 1550 | 474 | 1686 | 1367 | 594 | 432 |

*^a^* Chr, Chromosome; UN, Unknown.

**Table S4** Significant SNPs (*P* < 0.005), favourable alleles for ICT of yield, plants m^-2^, NDVI values, heads m^-2^, grains head^-1^, 1000 grain-weight and harvest index associated with tolerance to sodic-dispersive soil in wheat

| Traits | SNP^a^ | Chr^b^ | Year | Position | Allele | Effect |  |  |
| --- | --- | --- | --- | --- | --- | --- | --- | --- |
| Yield | scaffold40452;TaGBSv2-8437_3153139+11 | 1A | 2019 | 365186774 | C/T | 0.2328 |  |  |
|  | scaffold48187;TaGBSv2-210_8658523+63 | 1A | 2019 | 504980505 | G/A | 0.06 |  |  |
|  | scaffold108702;TaGBSv2-408_243146+42 | 1B | 2018, 2019 | 55014354 | C/T | 0.05 |  |  |
|  | scaffold9533;TaGBSv2-430_551402+111 | 1B | 2018, 2019 | 94828187 | A/G | 0.05 |  |  |
|  | scaffold9533;TaGBSv2-430_551402+94 | 1B | 2018, 2019 | 94828204 | T/G | -0.15 |  |  |
|  | scaffold9533;TaGBSv2-430_551402+85 | 1B | 2018, 2019 | 94828213 | G/C | -0.08 |  |  |
|  | scaffold9533;TaGBSv2-430_551402+21 | 1B | 2018, 2019 | 94828277 | A/G | 0.05 |  |  |
|  | scaffold47982-2;TaGBSv2-504_4891482+136 | 1B | 2018, 2019 | 411775338 | T/C | 0.07 |  |  |
|  | scaffold47982-2;TaGBSv2-504_4891482+110 | 1B | 2018, 2019 | 411775364 | G/C | 0.07 |  |  |
|  | scaffold47982-2;TaGBSv2-7772_4891456+77 | 1B | 2018, 2019 | 411775423 | C/T | 0.10 |  |  |
|  | scaffold128709;TaGBSv2-7503_60848+9 | 2B | 2018, 2019 | 34083794 | C/T | 0.05 |  |  |
|  | scaffold4776;TaGBSv2-8720_295746+143 | 3B | 2018, 2019 | 817580012 | T/C | 0.55 |  |  |
|  | scaffold4776;TaGBSv2-8720_295746+136 | 3B | 2018, 2019 | 817580019 | T/C | 0.55 |  |  |
|  | scaffold4776;TaGBSv2-8720_295746+89 | 3B | 2019 | 817580066 | G/A | 0.55 |  |  |
|  | scaffold126963;TaGBSv2-3527_999492+107 | 5A | 2019 | 5713954 | T/G | -0.02 |  |  |
|  | scaffold4266-1;TaGBSv2-3783_503841+127 | 5A | 2018, 2019 | 613704427 | T/C | -0.03 |  |  |
|  | scaffold90743;TaGBSv2-9900_986709+77 | 5A | 2018, 2019 | 614358933 | A/G | 0.07 |  |  |
|  | scaffold80421;TaGBSv2-11464_6484333+33 | 7B | 2019 | 148015309 | G/A | -0.17 |  |  |
|  | scaffold80421;TaGBSv2-11464_6484333+96 | 7B | 2019 | 148015372 | T/C | -0.17 |  |  |
| Plants m^-2^ | scaffold51925-2;TaGBSv2-6383_254739+74 | 1A | 2018, 2019 | 232828996 | A/G | -5.22 |  |  |
|  | scaffold108703;TaGBSv2-11630_1033239+176 | 1B | 2019 | 53737652 | A/T | 1.60 |  |  |
|  | scaffold108703;TaGBSv2-11630_1033239+112 | 1B | 2019 | 53737716 | C/G | 1.60 |  |  |
|  | scaffold115569;TaGBSv2-12139_3376643+153 | 1D | 2019 | 420202667 | C/T | -3.15 |  |  |
|  | scaffold46174;TaGBSv2-1020_4172344+81 | 2A | 2018, 2019 | 63307274 | G/A | -5.33 |  |  |
|  | scaffold46174;TaGBSv2-1020_4172344+91 | 2A | 2018, 2019 | 63307284 | C/T | -5.33 |  |  |
|  | scaffold46174;TaGBSv2-1020_4172344+172 | 2A | 2018, 2019 | 63307365 | A/T | -5.33 |  |  |
|  | scaffold46174;TaGBSv2-1020_4172344+181 | 2A | 2018, 2019 | 63307374 | G/A | -5.33 |  |  |
|  | scaffold46174;TaGBSv2-1020_4172344+190 | 2A | 2018, 2019 | 63307383 | C/T | -5.33 |  |  |
|  | scaffold41707;TaGBSv2-8390_16907212+27 | 3A | 2019 | 502921633 | G/A | -4.75 |  |  |
|  | scaffold41707;TaGBSv2-8390_16907212+28 | 3A | 2019 | 502921634 | C/T | -5.37 |  |  |
|  | scaffold41707;TaGBSv2-8390_16907212+173 | 3A | 2019 | 502921779 | C/T | -5.37 |  |  |
|  | scaffold7963;TaGBSv2-2477_2509891+57 | 3B | 2019 | 681510374 | C/T | 0.57 |  |  |
|  | scaffold10600;TaGBSv2-11023_3006486+120 | 6B | 2019 | 486517837 | A/T | -5.49 |  |  |
|  | scaffold133254;TaGBSv2-5745_334546+110 | 7B | 2019 | 484268231 | C/T | -0.21 |  |  |
|  | scaffold14497;TaGBSv2-5941_388420+60 | 7D | 2019 | 17321139 | A/G | -4.53 |  |  |
|  | scaffold53103;TaGBSv2-6090_4997923+165 | 7D | 2019 | 566251264 | G/C | 3.67 |  |  |
|  | scaffold53103;TaGBSv2-6090_4997923+64 | 7D | 2019 | 566251365 | A/T | -0.43 |  |  |
| NDVI | scaffold3010;TaGBSv2-1070_26733595+73 | 3B | 2019 | 211009958 | A/C | 0.01 |  |  |
|  | scaffold80997;TaGBSv2-7802_998336+156 | 3B | 2019 | 755524000 | C/T | 0.01 |  |  |
|  | scaffold2505-1;TaGBSv2-11139_1477483+90 | 6B | 2019 | 694681316 | A/G | 0.01 |  |  |
|  | scaffold21446;TaGBSv2-5534_51471+91 | 7A | 2019 | 696574393 | T/A | 0.01 |  |  |
|  | scaffold21446;TaGBSv2-5534_51471+177 | 7A | 2019 | 696574479 | G/A | 0.01 |  |  |
| Heads m^-2^ | scaffold92463;TaGBSv2-865_5008637+37 | 1D | 2019 | 356142294 | C/G | 7.98 |  |  |
|  | scaffold92463;TaGBSv2-865_5008637+66 | 1D | 2019 | 356142323 | T/C | -7.89 |  |  |
|  | scaffold92463;TaGBSv2-865_5008637+113 | 1D | 2019 | 356142370 | G/A | -7.89 |  |  |
|  | scaffold92463;TaGBSv2-865_5008637+117 | 1D | 2019 | 356142374 | A/G | 7.98 |  |  |
|  | scaffold92463;TaGBSv2-865_5008637+165 | 1D | 2019 | 356142422 | T/C | -7.89 |  |  |
| Grains head^-1^ | scaffold128709;TaGBSv2-7503_60848+9 | 2B | 2019 | 34083794 | C/T | 0.72 |  |  |
|  | scaffold128709;TaGBSv2-7503_60848+101 | 2B | 2019 | 34083886 | G/A | 1.04 |  |  |
|  | scaffold15232;TaGBSv2-8033_708396+119 | 2D | 2019 | 557966612 | C/T | -0.18 |  |  |
|  | scaffold15232;TaGBSv2-8033_708396+90 | 2D | 2019 | 557966641 | G/C | 3.19 |  |  |
|  | scaffold15232;TaGBSv2-8033_708396+8 | 2D | 2019 | 557966723 | G/T | -0.18 |  |  |
|  | scaffold73736;TaGBSv2-10500_525827+131 | 5D | 2019 | 437358882 | T/A | 3.03 |  |  |
|  | scaffold73736;TaGBSv2-10500_525827+103 | 5D | 2019 | 437358910 | G/A | 3.03 |  |  |
| 1000 grain-weight | scaffold13203;TaGBSv2-6443_270561+88 | 1A | 2019 | 588006280 | T/- | -1.60 |  |  |
|  | scaffold73775;TaGBSv2-8612_5252630+77 | 3B | 2019 | 658270393 | G/A | -2.94 |  |  |
|  | scaffold798;TaGBSv2-4960_5619817+76 | 6B | 2019 | 638828274 | C/T | 0.61 |  |  |
|  | scaffold798;TaGBSv2-11068_4095596+100 | 6B | 2019 | 640352471 | G/A | 1.29 |  |  |
|  | scaffold798;TaGBSv2-11068_4095596+55 | 6B | 2019 | 640352516 | A/G | 0.78 |  |  |
| Harvest index | scaffold139507;TaGBSv2-6393_1147474+41 | 1A | 2019 | 555622815 | C/A | 0.04 |  |  |
|  | scaffold13203;TaGBSv2-6443_270561+88 | 1A | 2019 | 588006280 | T/- | -0.04 |  |  |
|  | scaffold128709;TaGBSv2-7503_60848+9 | 2B | 2018, 2019 | 34083794 | C/T | 0.02 |  |  |
|  | scaffold128709;TaGBSv2-7503_60848+101 | 2B | 2018, 2019 | 34083886 | G/A | 0.02 |  |  |
|  | scaffold158337;TaGBSv2-7509_168378+155 | 2B | 2018, 2019 | 41415831 | T/C | -0.01 |  |  |
|  | scaffold73736;TaGBSv2-10500_525827+131 | 5D | 2019 | 437358882 | T/A | A | | 0.06 |
|  | scaffold73736;TaGBSv2-10500_525827+103 | 5D | 2019 | 437358910 | G/A | A | | 0.06 |
|  | scaffold100418;TaGBSv2-10929_6521163+26 | 6B | 2019 | 69712612 | C/T | C | | 0.02 |
|  | scaffold100418;TaGBSv2-10929_6521163+108 | 6B | 2019 | 69712694 | C/T | C | | 0.01 |
|  | scaffold100418;TaGBSv2-10929_6521163+170 | 6B | 2019 | 69712756 | T/C | C | | 0.04 |
|  | scaffold16066;TaGBSv2-5371_2584168+121 | 7A | 2019 | 177135543 | G/A | A | | 0.03 |
|  | scaffold16066;TaGBSv2-5371_2584168+49 | 7A | 2019 | 177135615 | T/C | C | | 0.03 |

*^a^* SNP, Single nucleotide polymorphism; *^b^* Chr, Chromosome. Data for heads m^-2^, grains head^-1^, 1000 grain-weight for 2018 trial are not available.
